# Effect of Preoperative Thoracic Paravertebral Blocks on Emergence Agitation During Tracheal Extubation: A Randomized Controlled Trial

**DOI:** 10.3389/fmed.2022.902908

**Published:** 2022-06-22

**Authors:** Wei Liu, Taijun Luo, Fei Wang, Ding Zhang, Tao Liu, Jiapeng Huang, Shaofa Xu

**Affiliations:** ^1^Department of Anesthesiology, Beijing Chest Hospital, Capital Medical University, Beijing, China; ^2^Department of Anesthesiology, Beijing Jishuitan Hospital, Beijing, China; ^3^Department of Anesthesiology and Perioperative Medicine, University of Louisville, Louisville, KY, United States; ^4^Department of Anesthesia, Jewish Hospital, Louisville, KY, United States; ^5^Department of Thoracic Surgery, Beijing Chest Hospital, Capital Medical University, Beijing, China

**Keywords:** emergence agitation, thoracoscopic, thoracic paravertebral block, intercostal nerve blocks, randomized controlled trial

## Abstract

**Objective:**

This study aims to compare the effects of preoperative thoracic paravertebral blocks (TPVB) with intercoastal nerve blocks (ICNB) on emergence agitation (EA) during tracheal extubation in patients who underwent thoracoscopic lobectomy.

**Design, Setting, and Participants:**

A randomized clinical trial was conducted in patients undergoing thoracoscopic lobectomy at Beijing Chest Hospital between June 2019 and December 2020.

**Interventions:**

Patients were randomly assigned 1:1 to receive either ultrasound-guided preoperative TPVB or ICNB.

**Main Outcomes and Measures:**

The primary outcome was the occurrence of emergency agitation, which was evaluated by Aono’s four-point scale (AFPS). Secondary outcomes included hemodynamics [mean arterial pressure (MAP) and heart rate (HR)]; and post-operative pain intensity [visual analog scale (VAS), Ramsay sedation score (RSS), and patient-controlled analgesia (PCA) demand times].

**Results:**

Among the 100 patients aged 55–75 years old, 50 were randomized to each group; 97 patients completed the trial. Compared to the ICNB group, the occurrence of EA in the TPVB group was significantly lower [31.3% (15/48) vs. 12.2% (6/49), relative risk = 1.276, 95% CI: 1.02–1.60, *P* = 0.028]. For patients in the TPVB group, the MAP and HR at 5, 10, and 30 min after extubation were significantly lower; the intraoperative details including emergence time, extubation time, and consumption of sufentanil were significantly shorter than that in the ICNB group. Additionally, patients in the TPVB group showed significantly lower VAS at rest or coughing and significantly lower RSS at 60 and 240 min after extubation than patients in the ICNB group (all *P* < 0.05).

**Conclusion:**

Preoperative TPVB was associated with less EA during tracheal extubation when compared with ICNB in patients undergoing thoracoscopic lobectomy.

**Clinical Trial Registration:**

[http://www.chictr.org.cn/index.aspx], identifier [ChiCTR1900023852].

## Introduction

Emergence agitation (EA) in adults is manifested as psychomotor excitement with purposeless thrashing, restlessness, and disorientation, and is a common complication in the waking stage of general anesthesia. It is commonly seen in thoracoscopic lobectomy (1) and the occurrence might reach 19% in non-cardiac surgery (2). EA may cause self-extubation or accidental removal of catheters, cardiac-cerebral vascular events (3), or even death (4). Multiple factors have been demonstrated to contribute to EA such as acute pain, urinary catheterization, tracheal intubation, and psychological stress (5). Previous studies had identified that one of the highest risk factors is post-operative acute pain (6–8). Intravenous opioids can reduce the occurrence of EA, but those drugs may lead to a high occurrence of drowsiness, dizziness, nausea, vomiting, delayed recovery, respiratory depression, and chest wall muscle rigidity (9). Therefore, approaches to prevent EA are important to identify.

The intercostal nerve block is a convenient and effective method to improve post-operative pain in thoracic surgery and has been widely used in clinical practices. Previous studies showed that intrapleural intercostal nerve block with mini-thoracotomy could reduce the post-operative pain and contribute to improving post-operative outcomes after major pulmonary resections (10, 11).

Thoracic paravertebral analgesia is similar to epidural analgesia with fewer side effects and is increasingly utilized in clinical practices (12). Studies have shown that a two-point paravertebral injection can spread the solution to a wider area and is more effective than single-point injection for analgesia (13). Due to significant difficulties in defining the paravertebral space (PVS), the failure rate of the traditional approach is around 10.1% (14). With the rapid advancement of ultrasound use in recent years, ultrasound-guided, thoracic paravertebral block (TPVB) has been shown to increase the success rate of block significantly (15). Several systemic reviews suggested that both TPVB and intercostal nerve blocks (ICNB) could play a role in ameliorating post-thoracotomy pain (16–18).

This study aims to compare the effects of preoperative thoracic paravertebral blocks (TPVB) with intercoastal nerve blocks (ICNB) on EA during tracheal extubation in patients who underwent thoracoscopic lobectomy.

## Materials and Methods

### Study Design and Participants

Ethical approval for this trial was obtained from the Institutional Review Board of Beijing Chest Hospital [ID: (2018) Ethical Review of Clinical Trial Recommended Project No. 03-01]. Written informed consents were obtained from all participants.

This randomized controlled trial was conducted on patients who underwent thoracoscopic lobectomy at Beijing Chest Hospital between June 2019 and December 2020. The inclusion criteria were (1) patients with American Society of Anesthesiologists (ASA) physical status I–III undergoing thoracoscopic lobectomy; (2) conscious and able to independently describe and evaluate pain after explanations; (3) 55–75 years old. Exclusion criteria included (1) serious cardiopulmonary diseases; (2) serious coagulation/hepatorenal function disorders; (3) history of alcohol or drug abuse; and (4) body mass index (BMI) ≥ 40 kg/m^2^; (5) preoperative narcotic analgesic use history, (6) with acute or chronic pain or oral analgesics before surgery; and (7) mental disorders or cognitive impairment [the score assessed by Montreal Cognitive Assessment (MoCA) is less than 26 points] (19).

### Randomization

Eligible participants were randomly assigned into two groups at a 1:1 ratio *via* a computer-generated random number table randomization system for clinical research. All peri-operative assessments and data collection were accomplished by the nurses.

### Interventions

l Thoracic paravertebral blocks and ICNBs were performed in a lateral position under real-time ultrasound-guided out-of-plane technique. A Philips ultrasound machine (Philips, CX-50, Holland) with a high-frequency linear transducer (L12-3) was utilized. In the TPVB group, the ultrasonic transducer was positioned to the long axis of the thoracic spine which was approximately 2–3 cm lateral to the spinous process. Then, the ultrasound transducer was moved until the tip of the transverse process was visible. In this position, the thoracic PVS was visualized between two transverse processes and parietal pleura. The parasagittal out-of-plane technique of needle insertion that was initially introduced into anesthesia by Hara (20) was applied in our study. The position of needle insertion was located and marked. After skin infiltration with 2% lidocaine (1–3 ml), and a 22-gauge insulated regional block needle (Stimuplex D, B. Braun, Germany) was inserted out-of-plane from lateral to medial direction. Hydrolocation with sterile saline was utilized to identify the needle tip until anterior pleural deflection was visualized on ultrasound, indicating that the needle tip was located in the PVS. Patients in the TPVB group received 2 injections of the PVB at the T3-4 and T6-7 (T means thoracic vertebrae; 3–4 and 6–7 means entry points on thoracic vertebrae of the needle tip) PVS with 7.5 ml of 0.75% ropivacaine and 2.5 ml of 2% lidocaine. Fifteen minutes after the block administration, the pinprick method at the midclavicular line was used to assess the extent of the dermatomal blockade.

Patients in the ICNB group received intercostal nerve blocks depending on the locations of skin incisions (the fourth intercostal space in the anterior axillary line) and chest tube placement (the seventh intercostal space in the midaxillary line). Ultrasound was used to identify anatomic landmarks. Ribs were identified as hyperechoic streaks and the pleura appeared as hyperechoic lines between and below the ribs. A mixture of 7.5 ml of 0.75% ropivacaine and 2.5 ml of 2% lidocaine was injected at the level of incision using a 22-gauge nerve block needle in an out-of-plane fashion. The needle was advanced until the distal tip was immediately adjacent to the pleura. A tissue plane between the internal and innermost intercostal muscles was delineated as the local anesthetic agent.

All nerve blocks were administered by the same experienced anesthesiologists, and the same total dose of ropivacaine and lidocaine was administrated to patients in both groups. All complications were recorded, including pneumothorax, local anesthetic intoxication, and total spinal anesthesia. TPVB failure was diagnosed as two unblocked adjacent dermatomes during pinprick assessment.

Standard anesthesia monitoring was performed after patients entered the operation room, including pulse oxygen saturation, electrocardiogram, bispectral index (BIS) monitor, and invasive blood pressure in the radial artery.

No patient received any premedication. Anesthesia was induced with midazolam (0.05 mg/kg), sufentanil (0.1–0.2 mcg/kg), plasma target-controlled infusion (TCI) Marsh model (TCI propofol) (3.0–3.5 mcg/mL); and cisatracurium (0.3 mg/kg). Left or right double-lumen tube was used to intubate and ventilate mechanically with 100% oxygen. The flow was set at 3 L/min, respiratory rate at 12 breaths per min, I:E ratio of 1:1.5, and tidal volume at 6–8 ml/kg. Intermittent administration of sufentanil and TCI propofol 1.5–3.5 mcg/mL was used to keep the BIS between 40 and 60, and the systolic arterial pressure and heart rate (HR) within ± 20% of baseline values during the procedure. Cisatracurium was administered for muscle relaxation as required. The neuromuscular blockade was reversed and the patient was extubated if awake. After extubation, patients had intravenous patient-controlled analgesia (PCA) *via* an infusion pump to deliver oxycodone at an infusion rate of (16–18) mcg/kg/h and a bolus dose of 1.5 mg with a lockout time of 15 min during the first 48 h postoperatively.

### Outcomes

The primary outcome was the occurrence of EA which was evaluated using Aono’s four-point scale (AFPS) score. Patients’ status was divided into four stages by AFPS score (2, 21): stage 1, calm; stage 2, not calm but could be easily calmed; stage 3, not easily calmed, moderately agitated or restless; stage 4, combative, excited, or disoriented. EA was defined as an AFPS score ≥ 3 from “time zero” to 2 min after extubation.


$$E\,A\,o\,c\,c\,u\,r\,r\,e\,n\,c\,e=\frac{N\,u\,m\,b\,e\,r\,o\,f\,p\,a\,t\,i\,e\,n\,t\,s\,\left(s\,t\,a\,g\,e\,3+s\,t\,a\,g\,e\,4\right)}{T\,o\,t\,a\,l\,n\,u\,m\,b\,e\,r\,o\,f\,p\,a\,t\,i\,e\,n\,t\,s}\times 100\%$$

The secondary outcomes included intraoperative mean arterial pressure (MAP) and HR at different time points after extubation; blood loss; duration of anesthesia; duration of surgery; duration of one-lung ventilation (OLV); emergence time; extubation time; sufentanil consumption; and propofol consumption; and post-operative pain intensity was measured by visual analogue scale (VAS), Ramsay sedation score (RSS), and PCA demand times at different time points after extubation.

Emergence time was defined as the time between propofol termination and first eye opening. Extubation time was defined as the time between propofol termination and extubation. The MAP and HR was recorded at the end of surgery just before extubation, and at 5, 10, 30, and 60 min after extubation. VAS pain scores, RSS, and PCA demand times were recorded at 60 min, 240 min, 24 h, and 48 h after extubation.

### Statistical Analysis

The sample size was calculated based on a preliminary pre-experimental study using PASS 15.0 software (NCSS, Kaysville, UT, United States). The incidence of EA (RSS ≥ 1) in thoracoscopic lobectomy in our hospital was 45%. Assuming that at a significance level of 5%, the incidence in the TPVB group was reduced to 20%, the trial power at 80%, and the dropout rate is at 10–15%, the study required a minimum of 47 patients per group. This study expanded the sample size to 50 patients per group.

Statistical analyses were performed using SPSS software version 15 (SPSS, Inc., Chicago, IL, United States). The primary efficacy data on the occurrence of EA were examined using intention-to-treat analysis. The normality of distribution was assessed with a Q-Q plot and the Shapiro–Wilk test. Data conforming to normal distribution were described as mean ± standard deviation (SD) and non-normal distribution of data was shown as median (IQR). The normally distributed variables, including demographic characteristics data, intraoperative and recovery data, were assessed by independent *t-*test. Non-parametric data were analyzed using the Mann–Whitney U-test and descriptive variables were evaluated using the Chi-square test or Fisher’s exact test. Hemodynamic data were compared by repeated measurement analysis of variance. Multivariable logistic regression analysis was applied to measure the relative risk of TPVB vs. ICNB on EA. *P*-value < 0.05 was considered statistically significant.

## Results

**FIGURE 1:**
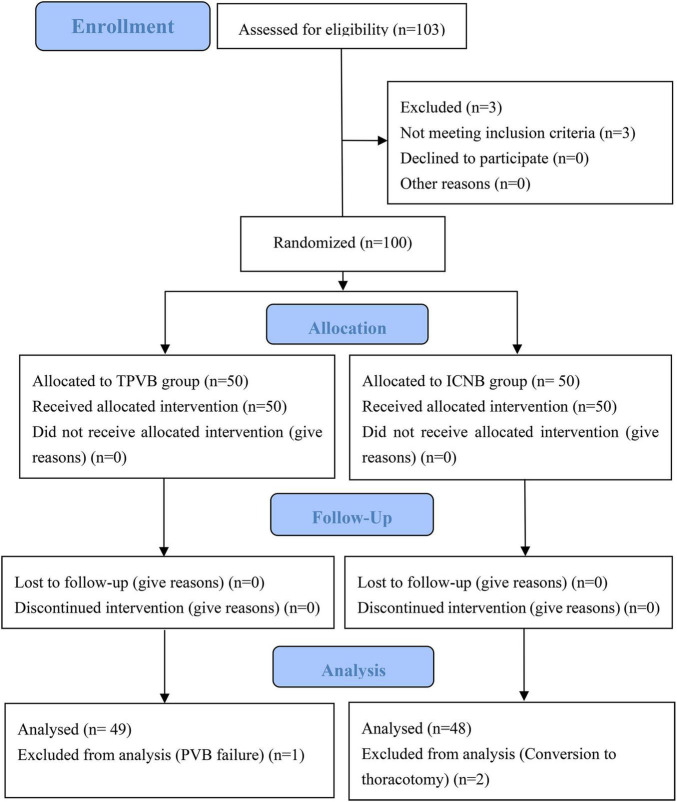
CONSORT flow diagram. TPVB indicates thoracic paravertebral blocks, and ICNB indicates intercoastal nerve blocks.

A total of 103 patients were assessed for eligibility. Among them, 3 patients did not meet the eligibility criteria and another 3 patients withdrew from the final analysis because of either conversion to thoracotomy or TPVB failure. Thus, 97 patients were included in the final analysis (Figure 1). There were no significant differences between the two groups in terms of age, gender, BMI, ASA classification, MoCA, lung function, preoperative forced expiratory volume in the first second (FEV1), preoperative forced vital capacity (FVC), preoperative FVC/FEV1 ratio and preoperative comorbidities (all *P* > 0.05) (Table 1).

**TABLE 1:** Clinical characteristics of the patients.

	TPVB group (*n* = 49)	ICNB group (*n* = 48)	*P*-value
Age (year)	62.4 ± 7.6	63.8 ± 7.6	0.360
Gender (male/female)	25/24	23/25	0.760
BMI (kg/m^2^)	25.1 ± 3.5	25.5 ± 3.7	0.528
ASA (I/II/III)	11/36/2	8/39/1	0.750
Montreal Cognitive Assessment	28.7 ± 1.4	28.9 ± 1.3	0.442
Lung Function			
Preoperative FEV_1_ (L)	2.33 ± 0.48	2.36 ± 0.54	0.816
Preoperative FVC (L)	3.01 ± 0.59	2.93 ± 0.75	0.545
Preoperative FEV_1_/FVC	78.06 ± 8.74	76.34 ± 10.18	0.375
Preoperative comorbidities			
Hypertension, *n*	21	22	0.768
Diabetes, *n*	10	10	0.959
Coronary heart disease, *n*	4	4	0.976

*BMI, body mass index; ASA (I/II/III), American Society of Anesthesiologists physical status I–III; FEV1, forced vital capacity rate of one second; FVC, forced vital capacity.*

The occurrence rates of EA during tracheal extubation in the TPVB group (12.2%, 6/49) were significantly less than in the ICNB group (31.3%, 15/48). Furthermore, multivariable logistic regression analysis suggested that preoperative TPVB (relative risk = 1.276, 95% CI: 1.020–1.600, *P* = 0.028) was associated with lower occurrence of EA (Table 2). However, the results of hemodynamics showed that the MAP and HR did not change significantly before extubation in both groups (*P* > 0.05), but were significantly lower in the TPVB group at 5, 10, and 30 min after extubation than those in the ICNB group (*P* < 0.05, Table 3). The emergence time (12.8 ± 7.2 vs. 16.1 ± 6.7, *P* = 0.023) and extubation time (14.4 ± 7.5 vs. 17.6 ± 6.9, *P* = 0.031) in TPVB group were significantly shorter than those in the ICNB group. Moreover, patients in the TPVB group showed less intraoperative consumption of sufentanil (26.1 ± 5.5 mcg vs. 29.2 ± 8.1 mcg, *P* = 0.028) than those in the ICNB group (Table 3).

**TABLE 2:** Comparison of Aono’s four-point scale between the two groups.

	AFPS stage ≥ 3	Relative Risk (95% CI)	*P*-value
TPVB group	6/49	1.276, (1.020 ∼ 1.600)	0.028
ICNB group	15/48	Ref.	Ref.

**TABLE 3:** Comparison of secondary outcomes between the two groups.

	TPVB group (*n* = 49)	ICNB group (*n* = 48)	*P*-value
**Hemodynamics**			
MAP (mmHg)			
Before extubation	92.2 ± 9.6	94.7 ± 10.4	0.225
5 min after extubation	96.0 ± 13.6	101.6 ± 12.9	0.041
10 min after extubation	92.3 ± 10.6	97.3 ± 13.1	0.047
30 min after extubation	88.8 ± 9.9	95.3 ± 11.7	0.004
60 min after extubation	88.4 ± 11.0	91.2 ± 9.9	0.194
HR (beats⋅min^–1^)			
Before extubation	69.9 ± 13.4	71.3 ± 13.8	0.615
5 min after extubation	88.0 ± 11.1	94.1 ± 12.0	0.011
10 min after extubation	84.9 ± 12.4	90.4 ± 11.7	0.026
30 min after extubation	80.0 ± 12.9	85.8 ± 12.2	0.022
60 min after extubation	78.6 ± 14.0	83.0 ± 11.0	0.087
**Intraoperative details and recovery characteristics**			
Blood loss (ml)	186 ± 156	212 ± 222	0.506
Duration of anesthesia (min)	187 ± 54	187 ± 55	0.995
Duration of surgery (min)	175 ± 55	172 ± 52	0.774
Duration of OLV (min)	165 ± 57	163 ± 52	0.872
Emergence time (min)	12.8 ± 7.2	16.1 ± 6.7	0.023
Extubation time (min)	14.4 ± 7.5	17.6 ± 6.9	0.031
Sufentanil (mcg)	26.1 ± 5.5	29.2 ± 8.1	0.028
Propofol (mg)	800 ± 240	820 ± 293	0.720
**Post-operative pain intensity**			
VAS score at rest			
60 min	0 (0–1)	1 (0–1)	0.161
240 min	0 (0–1)	0 (0–1.75)	0.001
24 h	1 (0–2)	1.5 (1–2)	0.018
48 h	1 (0–1)	1 (1–2)	0.028
**VAS score at coughing**			
60 min	1 (0–2)	2 (0–3)	0.039
240 min	1 (0–2)	2 (1–3)	0.014
24 h	2 (2–3)	3 (2–3.75)	0.014
48 h	2 (1–2.5)	2 (2–4)	0.022
**RSS scores**			
60 min	2 (2–3)	3 (2–3.75)	0.017
240 min	2 (2–2)	2 (2–3)	0.028
24 h	2 (2–2)	2 (2–2)	1.000
48 h	2 (2–2)	2 (2–2)	1.000
**PCA demand times**			
60 min	0 (0–1)	0 (0–1)	0.460
240 min	0 (0–1)	1 (0–2)	0.381
24 h	1 (1–3)	2 (0–4)	0.526
48 h	2 (1–4.5)	4 (0–6)	0.173

*MAP, mean arterial pressure; HR, heart rate; OLV, one-lung ventilation; VAS, visual analogue scale; RSS, Ramsay sedation score, PCA, patient-controlled analgesia.*

Further analysis showed that the post-operative pain was well controlled in both groups from 1 to 48 h after surgery (mean VAS pain scores less than 3). There were significantly lower VAS pain scores in the TPVB group at rest or coughing at each time point than those in the ICNB group (*P* < 0.05). Additionally, the RSS in the TPVB group at 60 and 240 min after extubation was significantly lower than those of the patients in the ICNB group (*P* < 0.05, Table 3). There were 5 cases of post-operative nausea in the TPVB group and 3 cases in the ICB group (*P* = 1.000) and there was no vomiting in either group.

## Discussion

This study showed that preoperative TPVB significantly decreased the occurrence of EA during tracheal extubation and the changes in hemodynamic instability after tracheal extubation in patients who underwent thoracoscopic lobectomy.

There might be several factors to explain the better efficacy of TPVB when compared to ICNB. First, the two-level injection of local anesthetics (LA) into the PVS at T_3–4_ and T_6–7_ can spread across from T_1_ to T_8_ (T1–T8 means the anesthetic plane). The two-level injection of LA into the intercostal space could only result in two dermatomal blockades. Second, TPVB might cause unilateral sympathetic and somatic nerve blockade, and preoperative ICNB could only cause segmental somatic nerve blockade. In addition, preoperative TPVB might reduce central sensitization by blocking the transference of nociceptive stimulation to the central nervous system. As a result, TPVB could decrease the occurrence of hyperalgesia and allodynia. The follow-up data also indicate that patients who received preoperative TPVB had lower VAS pain scores at rest and coughing than patients with ICNBs at each time point within 48 h after surgery. Thirdly, less use of sufentanil may avoid or reduce the hyperalgesia effects induced by opioids (22). Taken together, TPVB might reduce the occurrence of EA possibly by alleviating the acute post-operative pain. Previous studies showed that regional nerve blockade not only enhances post-operative analgesia but also produces a higher quality of recovery after surgery (23–25). This study found that patients receiving TPVB had a shorter emergence and extubation time than those with ICNBs. Furthermore, patients in the TPVB group presented a lower RSS within 4 h postoperatively than that in the ICNB group. Additionally, TPVB could reduce the general anesthetic dosage of sufentanil during operation. At the end of the surgery, the residual anesthetics in patients were lower and could be naturally eliminated more quickly, making them transition from the anesthesia state to a normal waking state.

EA can increase sympathetic nerve excitability, induce hemodynamic changes including hypertension and tachycardia, and may lead to cerebral vascular events. Patients undergoing lobectomy are usually older and complicated with multiple comorbidities, such as cardiovascular diseases, hypertension, and diabetes. Although there were no cardio-cerebral vascular events in either group, patients in the TPVB group maintained better hemodynamic stability when compared to the ICNB group during tracheal extubation. The hemodynamic stability in the TPVB group might be from the blockade of the cardio-accelerator nerves (T_1_–T_4_) with this block.

Vishal et al. (26) showed that the ultrasound-guided single-injection TPVB provided equivalent dermatomal spread and duration of analgesia when compared with the multiple-injection TPVB. The reason we chose two-level thoracic PVS injection was that thoracoscopic surgery is usually performed through two access ports in our hospital (27). The procedure port (4-cm incision) and the scope port (1.5-cm incision) were chosen at the level of the 3rd or 4th intercostal space in the anterior axillary line and the 7th or 8th intercostal space along the post-axillary line respectively, so the block area needs to cover T_3_–T_8_ unilaterally. Cowie et al. (28) showed that 20 ml of contrast dye could spread 4.5 segments after a single paravertebral injection and could spread to 6 segments with a two-level paravertebral injection in a cadaveric study. In addition, Kasimahanti et al. (13) reported that patients receiving two-level TPVB showed better analgesic effects than those with single-level TPVB. Copik (29) and Coopey (30) found that the ultrasound-guided TPVB failure rate in adults ranged from 3.9 to 7.4%. In this study, the failure rate was 2.00% (1/50) as one patient developed sensory blockade in less than 3 adjacent segments. The main cause of failure block might be the difficulty of clearly defining the transverse process (TP) with ultrasound due to excessive tissues.

There are some limitations to our study. First, the sample size was small and there might be a type 2 error. Second, previous studies indicated that the preoperative anxiety state was related to agitation and this study did not evaluate the preoperative anxiety state of these patients.

In conclusion, preoperative TPVB was associated with less EA during tracheal extubation when compared with ICNB in patients undergoing thoracoscopic lobectomy.

## Data Availability Statement

The original contributions presented in this study are included in the article/supplementary material, further inquiries can be directed to the corresponding author.

## Ethics Statement

The present study was approved by the Institutional Review Board of Beijing Chest Hospital [ID: (2018) Ethical Review of Clinical Trial Recommended Project No. 03-01]. The patients/participants provided their written informed consent to participate in this study.

## Author Contributions

WL: study design, data analysis, manuscript revision, and critical content editing. TJL: statistical review and manuscript revision. FW: data collection and manuscript preparation. DZ: data collection and manuscript revision. TL: data verification and manuscript revision. JH: study design and data analysis. SX: study design, data analysis, and critical content editing. All authors have read and agreed to the published version of the manuscript.

## Conflict of Interest

The authors declare that the research was conducted in the absence of any commercial or financial relationships that could be construed as a potential conflict of interest. The reviewer LZ declared a shared parent affiliation, with the authors, WL, FW, DZ, TL, and SX, to the handling editor at the time of the review.

## Publisher’s Note

All claims expressed in this article are solely those of the authors and do not necessarily represent those of their affiliated organizations, or those of the publisher, the editors and the reviewers. Any product that may be evaluated in this article, or claim that may be made by its manufacturer, is not guaranteed or endorsed by the publisher.
